# Characterization of Jamaican* Delonix regia* and* Cassia fistula* Seed Extracts

**DOI:** 10.1155/2016/3850102

**Published:** 2016-03-10

**Authors:** Andrea Goldson Barnaby, Raymond Reid, Vaughn Rattray, Ruth Williams, Marcel Denny

**Affiliations:** Department of Chemistry, The University of the West Indies, Mona, Kingston 7, Jamaica

## Abstract

*Delonix regia *and* Cassia fistula* seed extracts were evaluated for their antioxidant activity, total phenolics, ash, zinc and fatty acid content. Fourier Transform Infrared Spectroscopy (FTIR) was utilized to assess the chemical functionalities present within the seeds. Antioxidant activity was determined by the 2,2-diphenyl-1-picrylhydrazyl (DPPH) and Trolox equivalent antioxidant capacity (TEAC) assays. Total phenolics were determined by the Folin-Ciocalteu assay. Lipid extracts were characterized by nuclear magnetic resonance spectroscopy and gas chromatography/mass spectrometry. Zinc concentration was determined by atomic absorption spectroscopy. Extracts from the seeds of* C. fistula* had a higher antioxidant activity, free radical scavenging activity, and phenolic content than* D. regia*. FTIR revealed that the seeds are a rich source of protein with small quantities of fat.* C. fistula* extracts contained a higher percentage of total fat than* D. regia*. Palmitic acid was identified as the predominant saturated fatty acid in both extracts. Oleic acid and linoleic acid were identified in smaller quantities. Seed extracts may be considered for use in food and nutraceutical applications.

## 1. Introduction

*Delonix regia *and* Cassia fistula *can be found interspersed throughout the island of Jamaica and are primarily ornamental in nature. There is limited knowledge regarding the chemical composition of the seeds from trees grown in Jamaica.* D. regia*, Raffin (syn.* Poinciana regia*, Bojer ex Hook) also known as “flamboyant”, or “flame tree”, belongs to the Caesalpiniaceae family (family: Leguminosae, subfamily: Fabaceae). The tree is known to reach heights of approximately 12 m, whereas the flowers, which cover the tree crown, show colours ranging from orange to red. Carotenoids (*β*-carotene, lutein, rubixanthin, *β*-cryptoxanthin, and zeaxanthin) and anthocyanins (peonidin-3-O-glucoside, petunidin-3-o-acetyl-glucoside, cyanidin-3-O-rutinoside, and cyanidin-3-O-glucoside) are responsible for the vibrant colours observed in the petals of the flower [1]. In Africa, extracts of the flower are used to produce a traditional health beverage which contains the phenolic acids 3,4,5-trihydroxybenzoic (gallic acid), 3,4-dihydroxybenzoic (protocatechuic acid), and 2-hydroxy 5-[(3,4,5 trihydroxyphenyl) carbonyl oxy] benzoic acid [2].* D. regia* flower extract is known to also contain flavonols such as quercetin and its glycosides (quercetin-3-O-glucoside, quercetin-3-O-galactoside, rutin, quercetin-3-O-robinobioside, and quercetin trihexoside), as well as kaempferol rhamnosylhexoside and isorhamnetin rhamnosylhexoside [2].* Cassia fistula* L. (family: Leguminosae, subfamily: Fabaceae) is a semiwild Indian* Laburnum* that is widely cultivated in Mauritius but can also be found in Asia, Africa, Latin America, and the Caribbean. The tree has yellow flowers and is sometimes referred to as Golden Shower.* C. fistula* has been identified as a potentially novel source of free radical scavenging compounds [3]. Also in this case there is limited information on the chemical composition of extracts from the seeds of trees grown in Jamaica. In order to expand our knowledge on the chemical composition of seeds from these leguminous trees, chromatographic and spectroscopic techniques, as well as colorimetric methods, were applied. The antioxidant activity, total phenolics, fatty acid profile, and zinc composition of the seed extracts were evaluated. Seeds were also analyzed utilizing FTIR.

## 2. Materials and Methods

### 2.1. Plant Material

*D. regia* and* C. fistula* seed pods were randomly collected from trees located on the campus of the University of the West Indies, Kingston, Jamaica. Seeds were removed from mature dry pods. Ten pods represented one sample subset. Whole seeds were ground using a laboratory mill (30 sec at 25°C, Ika-Werke M20 Analytical Mill, Staufen, Germany).

### 2.2. The 1,1-Diphenyl-2-picrylhydrazyl (DPPH) Radical Scavenging Assay

The DPPH assay was performed according to the method of Brand-Williams et al. [4]. Samples (200 mg) were extracted with methanol (2 mL, 80%) containing hydrochloric acid (1%) at room temperature on an orbital shaker (200 rpm, Gallenkamp, England). Extracts were centrifuged (3200 rpm, 10 min) and the resulting supernatant was diluted with methanol (1 : 3, 1 mL) and reacted with DPPH (0.004%, 1 mL, 30 min). The absorbance was measured at 517 nm using a spectrophotometer (Helios Omega, Thermo Fisher Scientific). A standard calibration curve was generated and the results were expressed as mg/g gallic acid equivalents. Samples were analyzed in triplicate. Data obtained were useful to calculate the radical scavenging capacity according to the following formula:

(1) $$\begin{array}{c} \%=\left[\;1-\frac{A_{1}}{A_{0}}\;\right]*100, \end{array}$$

where *A*_1_ is absorbance of sample at 517 nm and  *A*_0_ is absorbance of control at 517 nm.

### 2.3. Total Phenolic Content

Total phenolics were determined using the Folin-Ciocalteu assay with modifications [5]. Samples (200 mg) were extracted with methanol (2 mL, 80%) containing hydrochloric acid (1%) at room temperature on an orbital shaker (200 rpm, Gallenkamp, England). Extracts were centrifuged (3200 rpm, 10 min) and the resulting supernatant (100 *μ*L) reacted with Folin-Ciocalteu reagent (10%, 750 *μ*L) and mixed for 5 min followed by addition of Na_2_HCO_3_ solution (7.5%, 750 *μ*L). The solution was incubated at 22°C (1.5 h) and the absorbance was measured at 760 nm using a spectrophotometer (Helios Omega, Thermo Fisher Scientific). A standard calibration curve of gallic acid (0–200 mg/L) was generated and the results were expressed as mg gallic acid/g.

### 2.4. Trolox Equivalent Antioxidant Capacity (TEAC) Assay

Free radical scavenging activity of methanolic extracts was also determined as Trolox equivalence [6]. 2,2′-Azinobis-(3-ethylbenzothiazoline-6-sulfonic acid) radical (ABTS•, 5 mM) was prepared in sodium phosphate buffer (7 mM, pH 7). Samples (20 *μ*L) were combined with ABTS• (2 mL) and the absorbance readings were recorded after 2 min at 750 nm. A standard calibration curve of Trolox (0–300 *μ*M) was prepared.

### 2.5. Lipid Extraction

Oil was Soxhlet extracted from the dried, milled seeds with petroleum ether (bp 80–100°C, reflux), and concentrated* in vacuo*. Percent crude fat (dry weight basis) was determined gravimetrically.

### 2.6. Methylation of Lipid Extracts

Soxhlet extracted oil samples (50 *μ*L) were* trans*methylated with methanol/acetyl chloride solution [7]. The resulting fatty acid methyl esters (FAMEs) were determined by gas chromatography-mass spectrometry (GC-MS).

### 2.7. Gas Chromatography-Mass Spectrometry

Methylated oil in hexane (1.0 *μ*L) was chromatographed on an HP6890 series Gas Chromatograph interfaced with an HP5973 Mass Selective Detector. Constituent FAMEs were eluted with helium carrier gas (flow rate 1 cm^3^/min) through a DB-VRX column (20 m × 0.18 mm i.d. × 1.0 *μ*m film thickness, Agilent, Santa Clara, CA) in an oven programmed at 60°C for 3 min and increased at a ramp rate of 10°C/min up to 250°C for 15 min. Samples were injected at 230°C while the detector was maintained at 250°C. Constituents were identified by matching the mass spectra, National Institute of Standards and Technology (NIST) library of mass spectra (match quality > 80%).

### 2.8. ^1^H NMR and ^13^C NMR Spectroscopy

^1^H NMR and ^13^C NMR characterization were performed on a Bruker BioSpin 500 MHz Spectrometer (Massachusetts, USA) at 500 MHz. A 5 mm probe was used for ^1^H NMR and ^13^C NMR experiments. Lipid extracts were run in deuterated chloroform (CDCl_3_) at 25°C, with tetramethylsilane as the internal standard while for phenolic extracts, deuterated acetone was utilized as solvent.

### 2.9. Iodine Value

Iodine values were calculated based on the FAME content and were calculated utilizing the formula:

(2) $$\begin{array}{c} Predicted\;\;IV=xC1+yC2+zC3. \end{array}$$

C1, C2, and C3 correspond to the relative percentage concentrations of unsaturated fatty acids (one, two, and three double bonds, resp.) whereas *x*, *y*, and *z* are coefficients (*x* = 1, *y* = 1.5, and *z* = 2.62) [8].

### 2.10. Atomic Absorption Spectroscopy

Samples (1.5 g) were ashed in a muffle furnace (600°C for 1.5 h) and the resulting ash dissolved in HCl (10 mL, AR), diluted with deionized water, and filtered. Measurements for zinc were made using a Perkin Elmer 2380 Flame Atomic Absorption Spectrophotometer system equipped with the corresponding hollow cathode lamp at the time of analysis. The following parameters were utilized: lamp current 10 mA, wavelength 214 nm, and slit width 0.7 nm, with flame type consisting of air/acetylene and stoichiometric fuel flow at 0.9 to 1.21 min^−1^. Stock solutions of zinc were made and standard calibration curves were prepared. Results are expressed as ppm.

## 3. Fourier Transform Infrared Spectroscopy

A Bruker Vector 22 Fourier Transform Infrared (FTIR) Spectrometer was utilized to record the infrared spectra of milled seed samples of* D. regia* and* C. fistula*. OPUS software was used to acquire and manipulate the spectral data.

### 3.1. Data Analyses

Samples were analyzed in triplicate. Means and standard deviations of the data were presented.

## 4. Results and Discussion

Interest in the chemical and physiological properties of extracts from* D. regia* and* C. fistula *continues to increase with recent papers documenting their potential health benefit and applicability. Galactomannan, a storage polysaccharide, was isolated from the seeds of* D. regia* [9] with potential use as a thickening and stabilizing agent in the food industry [10]. Sesquiterpene (E) nerolidol (38.0%) was detected as the major essential oil in flowers of the tree and phytol (16.1%) in the leaves of* C. fistula* [11]. The ethyl acetate extract from the flowers of* C. fistula* has shown antifeedant and larvicidal effects on insects. Rhein (1,8-dihydroxy-anthraquinone-2-carboxylic acid) was identified as the active component from the ethyl acetate extract [12].

## 5. Free Radical Scavenging Activity and Total Phenolics

Legumes are a source of natural antioxidants [13]. Their antioxidant activity is based on the presence of different classes of compounds which includes phenolic acids and their derivatives. Derivatives include, for example, flavanols, anthocyanins, tocopherols, and vitamin C [13]. In a study conducted by Amarowicz and Raab, it was found that lentil (*Lens culinaris*) and faba beans (*Vicia faba minor*) possessed high levels of antioxidant activity [14]. The dominant phenolics identified in extracts of red lentil were quercetin diglycoside, catechin, digallate procyanidin, and* p*-hydroxybenzoic acid [15].

Extracts from* C. fistula* have been used extensively in traditional Indian medicine. High levels of antioxidants and phenolics (proanthocyanidins and flavonoids) have been detected in the pods [16] with bark extracts being reported as possessing antidiabetic properties [17]. Leaf, flower, and bark extracts of* D. regia* also possess antioxidant and antimicrobial attributes [18].* D. regia* leaf extracts exhibit anti-inflammatory activity [19]. Whereas the phenolic composition of the flowers of* D. regia* has been previously reported [2], there is limited information regarding the phenolic content of the seeds.

The antioxidant activity of* D. regia* and* C. fistula* seed extracts was determined by the DPPH free radical scavenging and TEAC assays and total phenolics by the Folin-Ciocalteu assay. The DPPH assay is frequently utilized in assessing the antioxidant properties of extracts [20]. The DPPH free radical is stable and changes colour from violet to yellow upon the addition of a proton. This decrease in colour may be quantified spectrophotometrically.

Higher levels of free radical scavenging activity and total phenolics were detected in the seed extracts of* C. fistula* compared to* D. regia* (Table 1). The free radical scavenging activity of* C. fistula* was twice that observed in* D. regia*. For the TEAC assay, values of 0.93 ± 0.02 and 1.71 ± 0.08 mg Trolox/g were obtained for* D. regia* and* C. fistula* seed extracts, respectively, thereby further substantiating that seed extracts of* C. fistula* have higher levels of antioxidant activity when compared to* D. regia*. Tannins have been reported as being the main contributors to the free radical scavenging properties of legume extracts [15]. Phenolic compounds contribute to antioxidant activity by serving as potent hydrogen donors due to the hydroxyl functionality present. They are able to scavenge free radicals, chelate metals which serve as catalysts in the production of free radicals, activate antioxidant enzymes, and inhibit oxidases [21]. Antioxidants assist in protecting the body from oxidative damage which may be caused by reactive oxygen species and results in several diseases, for example, cancer and cardiovascular disease [22]. Seed extracts of* C. fistula* and* D. regia* may prove to be a valuable source of natural antioxidants and may be considered for use in nutraceutical applications.

## 6. Crude Lipid Extract, Fatty Acid Profile, and Iodine Value

*C. fistula* seed extracts contained higher levels of total lipids (8.22 ± 0.47%) compared to* D. regia* (1.41 ± 0.65%). Palmitic acid was identified as the predominant fatty acid in both seed extracts (Table 2). Prior studies on* D. regia* and* C. fistula* seeds grown in Rajasthan and Nigeria identified linoleic acid as the predominant fatty acid present [23–26]. Linoleic acid was only identified in small quantities.* C. fistula* extracts contained more minor fatty acids compared to* D. regia *(Table 3). The predicted iodine values of* D. regia* and* C. fistula* were 44 and 16, respectively. Lipid extracts from* C. fistula* are therefore expected to be more stable to oxidation than* D. regia*. *β* sitosterol (3.99 ± 1.08%) and benzyl alcohol (3.27 ± 0.48%) were also identified in seed extracts from* C. fistula.* Increased oil yields may be obtained by the utilization of a pressure based system for extraction. The oil extract of cowpeas (*Vigna unguiculata*) has been shown to possess antidiabetic properties [27]. Oil extracts from* C*.* fistula* and* D. regia *may be evaluated for potential medicinal activity.

## 7. ^**1**^H and ^**13**^C NMR Spectroscopic Data 

**Figure 1:**
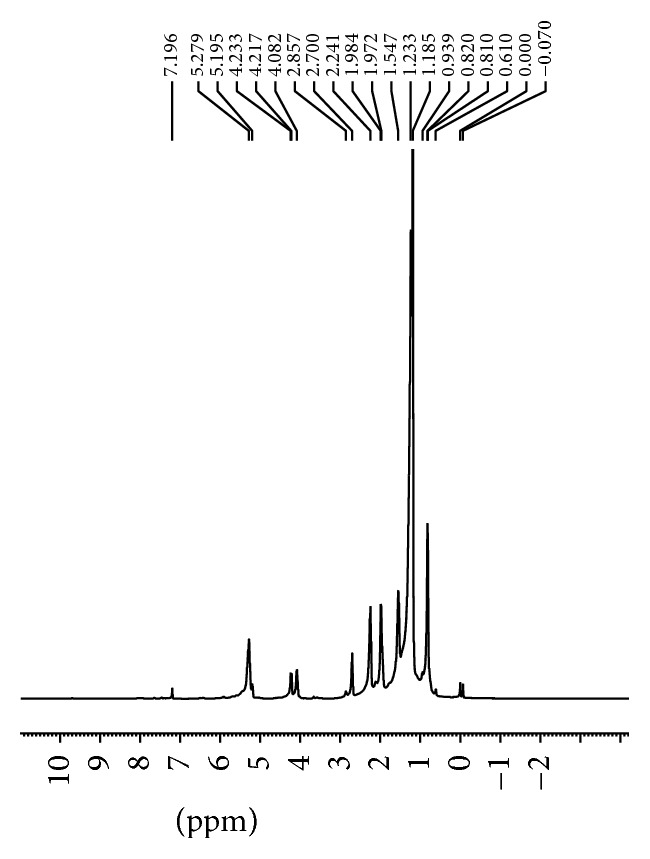
^1^H NMR of* C. fistula* lipid extracts.

**Figure 2:**
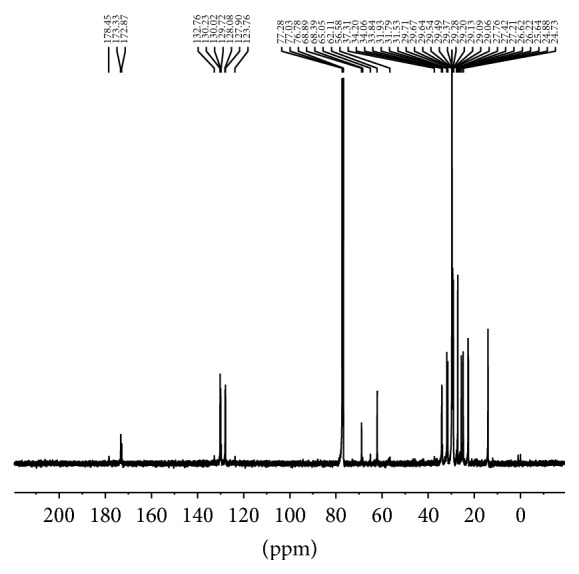
^13^C NMR of* C. fistula* lipid extracts.

^1^H NMR data (Figure 1) of the lipid extracts of the seeds revealed that the fatty acids present exist predominantly as triacylglycerols. This was indicated by two doublet of doublets (*δ* 4.15, *δ* 4.25) and a multiplet (*δ* 5.26) which are due to the protons on the triacylglycerol backbone (Table 4). Olefinic protons due to the presence of oleic acid and linoleic acid resonated at *δ* 5.34. Chemical shifts were observed at *δ* 2.75 (*D. regia*) and *δ* 2.70 (*C. fistula*) which are indicative of protons on a bis allylic carbon [28] due to the presence of linoleic acid. Chemical shifts for the fatty acid carbonyl bound to the 1,3-glycerol (*α*) and the 2-glycerol carbon (*β*) were observed at *δ* 172.65 and *δ* 173.09, respectively, in* D. regia* and *δ* 172.88 and *δ* 173.33 in* C. fistula* (Figure 2, Table 5). Unsaturation was demonstrated by chemical shifts occurring in the range of *δ* 128–130 ppm in the ^13^C NMR (Table 5). ^1^H NMR spectroscopic data of methanolic extracts showed evidence of a methyl group (*δ* 1.36), ether linkages (*δ* 3.63–*δ* 3.81), phenolics (*δ* 4.12), and vinylic protons (*δ* 5.35 and *δ* 5.42). The presence of the methyl and ether functionalities suggests that the phenolic compounds present are substituted. The vinylic protons resonated as doublets and are due to the aromatic ring of the phenolics. The ^13^C NMR spectra also confirmed the presence of vinylic carbons (*δ* 129.07 and *δ* 130.93). Carbons with hydroxyl functionalities were observed at *δ* 62.19, *δ* 63.38, *δ* 64.03, and *δ* 64.25. NMR spectroscopy is increasingly being utilized to determine sample authenticity [29–31].

## 8. Fourier Transform Infrared Spectroscopy

**Figure 3:**
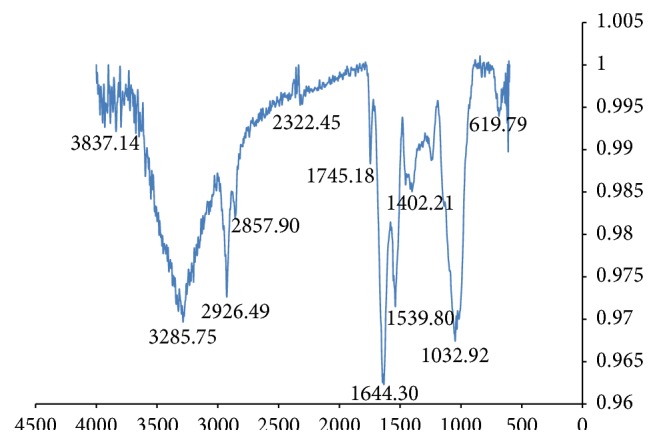
Analysis of* C. fistula* seeds utilizing FTIR.

**Figure 4:**
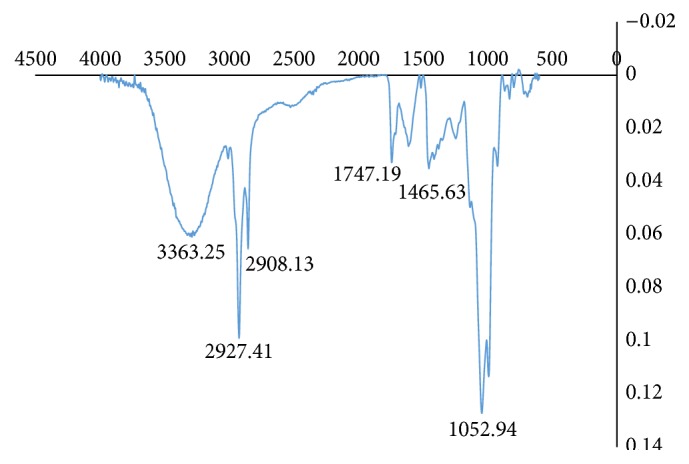
Analysis of* C. fistula* seed methanolic extracts utilizing FTIR.

FTIR is a rapid method of analysis which is also increasingly being utilized to detect adulteration in samples [32–35]. The FTIR spectroscopy profile for* C*.* fistula* and* D. regia* was similar (Table 6, Figure 3). The major bands observed are due to the high protein content of the seeds. Bands observed at 3270.37 cm^−1^ (*D. regia*) and 3285.75 cm^−1^ (*C. fistula*) are indicative of N-H stretching vibrations present in the amide functionality of proteins. Amide N-H bending vibrations were observed at 1542.60 cm^−1^ (*D. regia*) and 1539.80 cm^−1^ (*C. fistula*). For secondary amides, the N-H bending vibrations were observed at 1238.54 cm^−1^ and 1243.64 cm^−1^ [36]. Bands at 1399.98 cm^−1^, 1402.21 cm^−1^, 1645.96 cm^−1^, and 1644.30 cm^−1^ were due to the carbonyl functionality of the amides. The appearance of these strong bands indicates the presence of protein in the solid state [36]. Bands observed at 1455.10 cm^−1^ (*C. fistula*) and 1456.76 cm^−1^ (*D. regia*) were due to bending deformation of C-H vibration with stretching vibrations being observed at 2852.90 cm^−1^, 2922.57 cm^−1^, 2857.90 cm^−1^, and 2926.49 cm^−1^. Other pronounced bands were observed at 1742.63 cm^−1^ (*D. regia*) and 1745.18 cm^−1^ (*C. fistula*) due to the carbonyl stretching vibrations of triacylglycerols present [36]. FTIR spectroscopy of methanolic extracts of the seeds revealed a broad band at 3384.45 cm^−1^ (*D. regia*) and 3363.25 cm^−1^ (*C. fistula*) which is due to the hydroxyl functionality of the phenolics present (Figure 4). The C-H stretch and C-C stretch due to the aromaticity of phenolics were also observed (Table 7). Bands at 1747.19 cm^−1^ (*C. fistula*) and 1720.19 cm^−1^ are indicative of the presence of a carbonyl substituent on the phenolic ring. Sharp bands at 1052.94 cm^−1^ (*C. fistula*) and 1049.09 cm^−1^ (*D. regia*) also indicate that the phenolics present may be further substituted producing the carbon-oxygen stretching vibration (Table 7).

## 9. Zn Composition 

Seed extracts were found to contain 3.83 ± 0.60% (*D. regia*) and 5.48 ± 0.05% (*C. fistula*) ash. Zinc is an essential trace element which has antioxidant [37] and anti-inflammatory properties [38]. The ability of zinc to retard oxidative processes has been recognized for several years [39]. Seed samples were therefore analyzed for their zinc composition.* C. fistula* and* D. regia* seed extracts were found to contain similar concentrations of zinc, 34.5 ± 0.3 ppm and 38.4 ± 1.0 ppm, respectively.

## 10. Conclusion

The composition of* D. regia* and* C. fistula* seeds was investigated in the form of powder, ash, oil, phenolics, and antioxidant activity. The results indicate that the seeds are a rich source of proteins with fats and starch being present in small quantities. The seeds are also a source of antioxidants. This study further substantiates that extracts from the seeds of these trees may be of commercial value and warrant further investigation.

## Figures and Tables

**Table 1:** Antioxidant activity and total phenolic content of *D*.* regia* and *C. fistula* extracts expressed as gallic acid equivalents (GAE).

Parameters investigated	*D. regia*	*C. fistula*
Antioxidant activity (mg/g)	0.52 ± 0.02	1.15 ± 0.07
Total phenolics (mg/g)	1.54 ± 0.16	2.10 ± 0.26
Free radical scavenging activity (%)	15.30 ± 0.70	38.27 ± 1.25

**Table 2:** Fatty acid profile of *D. regia* and *C. fistula* seed extracts.

Fatty acid		*D. regia* %	*C. fistula* %
Palmitic acid	C16:0	41.59 ± 3.20	34.44 ± 6.39
Stearic acid	C18:0	24.10 ± 5.11	10.00 ± 0.93
Oleic acid (omega 9)	C18:1	24.75 ± 3.75	6.46 ± 0.54
Linoleic acid (omega 6)	C18:2	12.82 ± 2.11	6.29 ± 0.32

**Table 3:** Minor fatty acids identified in *C. fistula*.

Fatty acid		Percent composition
Pentadecylic acid	C15:0	0.38 ± 0.03
Palmitoleic acid	C16:1	0.31 ± 0.08
Margaric acid	C17:0	0.62 ± 0.10
Arachidic acid	C20:0	4.65 ± 0.18
Heneicosylic acid	C21:0	0.83 ± 0.09
Behenic acid	C22:0	5.75 ± 0.83
Lignoceric acid	C24:0	4.20 ± 0.07
Pentacosylic acid	C25:0	1.04 ± 0.01
Cerotic acid	C26:0	1.19 ± 0.12

**Table 4:** ^1^H Nuclear magnetic resonance spectroscopy data of seed lipid extracts.

Proton	Functionality	*D. regia* *δ* (ppm)	*C. fistula* *δ* (ppm)
CH_3_	Terminal methyl	0.86	0.81
CH_2_	Methylene	1.28	1.23
CH_2_-CH_2_-COO	All acyl chains	1.59	1.55
CH_2_-CH=CH	All unsaturated fatty acids	2.01	1.98
CH_2_-COO	All acyl chains	2.27	2.24
C=C-CH_2_-C=C	Protons attached to bis allylic carbon	2.73	2.70
CH_2_O(*α*)	Glycerol (triglycerides)	4.15	4.08
CH_2_O(*α*)	Glycerol (triglycerides)	4.25	4.23
CHO(*β*)	Glycerol (triglycerides)	5.26	5.20
CH=CH	Olefinic protons	5.34	5.28

**Table 5:** ^13^C Nuclear magnetic resonance spectroscopy data of seed lipid extracts.

Carbon	Assignment	*D. regia* *δ* (ppm)	*C. fistula* *δ* (ppm)
*α*-CH_3_	Acyl chains	13.88	13.85
*β*-CH_3_	Acyl chains	22.54	22.70
C3	Acyl chains	24.78	24.72, 24.88
C11	Diallylic	25.58	25.64
C8–11 (oleyl)	Allylic	27.12	26.62
C8–14 (linoleyl)	Allylic	27.12	27.21; 27.42, 27.75
CH_2*n*_	Acyl chains	29.01–29.56	29.06–29.71
C16	Linoleyl	31.42	31.53, 31.79, 31.93
*α*-C2	Acyl chains	33.83	33.84
*β*-C2	Acyl chains	33.97	34.06, 34.20, 37.31
*α*-CH_2_O	Glycerol moiety	62.06	62.11
*α*-CH_2_O	Glycerol moiety	64.98	65.06
*β*-CH_2_O	Glycerol moiety	69.01	68.89
C12	Linoleyl	127.87	127.90
C13	Linoleyl	128.05	128.08
C9	Oleyl	129.61	129.72
C10	Oleyl	129.91	130.02
C10	Linoleyl	130.11	130.23, 132.76
*α*-C1	Glycerol moiety	172.65	172.88
*β*-C1	Glycerol moiety	173.09	173.33
C1	Free fatty acid	178.20	178.45
C1	Free fatty acid	178.36	

**Table 6:** FTIR spectroscopic data of seed samples of *D. regia* and *C. fistula*.

Functionality	*D. regia* cm^−1^	*C. fistula* cm^−1^
C-O-C (ether functionality, starch)	995.69	1032.92, 1047.46
N-H bending (secondary amide)	1238.54	1243.64
C=O bending vibration (primary amide)	1399.98	1402.21
C-H vibration (bending deformation)	1456.76	1455.10
N-H bending (amide II)	1542.60	1539.80
C=O (amide I)	1645.96	1644.30
C=O stretching vibrations (lipids)	1742.63	1745.18
CH stretching vibrations (Symmetric & asymmetric)	2852.90, 2922.57	2857.90, 2926.49
N-H stretching vibration (amide) & OH stretching vibrations (starch)	3270.37	3285.75

**Table 7:** FTIR spectroscopic data of methanolic extracts of seed samples of *D. regia* and *C. fistula*.

Functionality	*D. regia* cm^−1^	*C. fistula* cm^−1^
C-O stretch	1049.09	1052.94
C-C stretch (aromatic ring)	1421.28	1465.63
C-C stretch (aromatic ring)	1619.91	1633.41
C=O stretching vibrations	1720.19	1747.19
C-H stretch (aromatic ring)	2908.13	2908.13
C-H stretch (aromatic ring)	2927.41	2927.41
O-H stretch (phenolics)	3384.45	3363.25

## References

[1] Veigas J. M., Divya P., Neelwarne B. (2012). Identification of previously unreported pigments among carotenoids and anthocyanins in floral petals of *Delonix regia* (Hook.) Raf. *Food Research International*.

[2] Adjé F. A., Lozano Y. F., Le Gernevé C. (2012). Phenolic acid and flavonol water extracts of *Delonix regia* red flowers. *Industrial Crops and Products*.

[3] Bhalodia N. R., Acharya R. N., Shukla V. J. (2012). Evaluation of *in vitro* antioxidant activity of hydroalcoholic seed extratcs of *Cassia fistula* Linn. *Free Radicals and Antioxidants*.

[4] Brand-Williams W., Cuvelier M. E., Berset C. (1995). Use of a free radical method to evaluate antioxidant activity. *LWT—Food Science and Technology*.

[5] Singleton V. L., Rossi J. A. (1965). Colorimetry of total phenolics with phosphomolybdic-phosphotungstic acid reagents. *American Journal of Enology and Viticulture*.

[6] Seeram N. P., Aviram M., Zhang Y. (2008). Comparison of antioxidant potency of commonly consumed polyphenol-rich beverages in the United States. *Journal of Agricultural and Food Chemistry*.

[7] Masood A., Stark K. D., Salem N. (2005). A simplified and efficient method for the analysis of fatty acid methyl esters suitable for large clinical studies. *Journal of Lipid Research*.

[8] Kyriakidis N. B., Katsiloulis T. (2000). Calculation of iodine value from measurements of fatty acid methyl esters of some oils: comparison with the relevant American Oil Chemists' Society method. *Journal of the American Oil Chemists' Society*.

[9] Tamaki Y., Teruya T., Tako M. (2010). The chemical structure of galactomannan isolated from seeds of *Delonix regia*. *Bioscience, Biotechnology and Biochemistry*.

[10] Pacheco-Aguirre J., Rosado-Rubio G., Betancur-Ancona D., Chel-Guerrero L. (2010). Physicochemical properties of carboxymethylated flamboyant (*Delonix regia*) seed gum. *CyTA—Journal of Food*.

[11] Tzakou O., Loukis A., Said A. (2007). Essential oil from the flowers and leaves of *Cassia fistula* L.. *Journal of Essential Oil Research*.

[12] Duraipandiyan V., Ignacimuthu S., Gabriel Paulraj M. (2011). Antifeedant and larvicidal activities of Rhein isolated from the flowers of *Cassia fistula* L.. *Saudi Journal of Biological Sciences*.

[13] Amarowicz R., Pegg R. B. (2008). Legumes as a source of natural antioxidants. *European Journal of Lipid Science and Technology*.

[14] Amarowicz R., Raab B. (1997). Antioxidative activity of leguminous seed extracts evaluated by chemiluminescence methods. *Zeitschrift für Naturforschung*.

[15] Amarowicz R., Estrella I., Hernández T. (2009). Antioxidant activity of a red lentil extract and its fractions. *International Journal of Molecular Sciences*.

[16] Luximon-Ramma A., Bahorun T., Soobrattee M. A., Aruoma O. I. (2002). Antioxidant activities of phenolic, proanthocyanidin, and flavonoid components in extracts of *Cassia fistula*. *Journal of Agricultural and Food Chemistry*.

[17] Daisy P., Saipriya K. (2012). Biochemical analysis of *Cassia fistula* aqueous extract and phytochemically synthesized gold nanoparticles as hypoglycemic treatment for diabetes mellitus. *International Journal of Nanomedicine*.

[18] Shabir G., Anwar F., Sultana B. (2011). Antioxidant and antimicrobial attributes and phenolics of different solvent extracts from leaves, flowers and bark of Gold Mohar [*Delonix regia* (Bojer ex Hook.) Raf]. *Molecules*.

[19] Shewale V. D., Deshmukh T. A., Patil L. S., Patil V. R. (2012). Anti-inflammatory activity of *Delonix regia* (Boj. Ex. Hook). *Advances in Pharmacological Sciences*.

[20] Zhou K., Yu L. (2004). Effects of extraction solvent on wheat bran antioxidant activity estimation. *LWT—Food Science and Technology*.

[21] Kulkarni A. P., Aradhya S. M., Divakar S. (2004). Isolation and identification of a radical scavenging antioxidant—punicalagin from pith and carpellary membrane of pomegranate fruit. *Food Chemistry*.

[22] Gerber M., Boutron-Ruault M.-C., Hercberg S., Riboli E., Scalbert A., Siess M.-H. (2002). Food and cancer: state of the art about the protective effect of fruits and vegetables. *Bulletin du Cancer*.

[23] Arora A., Sen R., Singh J. (2010). Fatty acid composition of *Delonix regia* (Gulmohar) seed oil from arid zone of Rajasthan. *Journal of the Indian Council of Chemists*.

[24] Arora A., Sen R. (2010). Determination of fatty acids in plant seeds of leguminosae family from arid zone of Rajasthan. *Asian Journal of Chemistry*.

[25] Adewuyi A., Oderinde R. A., Rao B. V. S. K., Prasad R. B. N., Anjaneyulu B. (2010). Chemical component and fatty acid distribution of *Delonix regia* and *Peltophorum pterocarpum* seed oils. *Food Science and Technology Research*.

[26] Adewuyi A., Oderinde R. A. (2011). Analysis of the mineral nutrient, chemical composition and distribution of fatty acids in the lipid classes of the seed oils of underutilized legumes from Nigeria. *Rivista Italiana delle Sostanze Grasse*.

[27] Ashraduzzaman M., Alam M. A., Khatun S., Banu S., Absar N. (2011). *Vigna unguiculata* linn. Walp. Seed oil exhibiting antidiabetic effects in alloxan induced diabetic rats. *Malaysian Journal of Pharmaceutical Sciences*.

[28] Thoss V., Murphy P. J., Marriott R., Wilson T. (2012). Triacylglycerol composition of British bluebell (*Hyacinthoides* non-scripta) seed oil. *RSC Advances*.

[29] Kamm W., Dionisi F., Hischenhuber C., Engel K.-H. (2001). Authenticity assessment of fats and oils. *Food Reviews International*.

[30] Lachenmeier D. W., Humpfer E., Fang F. (2009). NMR-spectroscopy for nontargeted screening and simultaneous quantification of health-relevant compounds in foods: the example of melamine. *Journal of Agricultural and Food Chemistry*.

[31] Capuano E., Lommen A., Heenan S., de la Dura A., Rozijn M., van Ruth S. (2012). Wild salmon authenticity can be predicted by 1H-NMR spectroscopy. *Lipid Technology*.

[32] Fadzlillah N. A., Che Man Y. B., Rohman A. (2014). FTIR spectroscopy combined with chemometric for analysis of sesame oil adulterated with corn oil. *International Journal of Food Properties*.

[33] Rohman A., Man Y. B. C. (2011). Palm oil analysis in adulterated sesame oil using chromatography and FTIR spectroscopy. *European Journal of Lipid Science and Technology*.

[34] Rohman A., Martsasi A., Riyanto S. (2013). Simultaneous quantitative analysis of red fruit oil and sesame oil using FTIR spectroscopy and multivariate calibrations. *International Food Research Journal*.

[35] Rohman A., Che Man Y. B. (2011). Application of gas chromatography and FTIR spectroscopy for analysis of palm oil in adulterated sesame oil. *European Journal of Lipid Science and Technology*.

[36] El-Bahy G. M. S. (2005). FTIR and Raman spectroscopic study of Fenugreek (*Trigonella foenum graecum* L.) seeds. *Journal of Applied Spectroscopy*.

[37] Bray T. M., Bettger W. J. (1990). The physiological role of zinc as an antioxidant. *Free Radical Biology and Medicine*.

[38] Prasad A. S. (2014). Zinc: an antioxidant and anti-inflammatory agent: role of zinc in degenerative disorders of aging. *Journal of Trace Elements in Medicine and Biology*.

[39] Powell S. R. (2000). The antioxidant properties of zinc. *Journal of Nutrition*.

